# The management of acute lower gastrointestinal bleeding using a Sengstaken-Blakemore tube

**DOI:** 10.1016/j.ijscr.2020.09.066

**Published:** 2020-09-21

**Authors:** Michael G. Fadel, Piers R. Boshier, Ann-Marie Howell, Mohamad Iskandarani, Paris Tekkis, Christos Kontovounisios

**Affiliations:** a Department of Colorectal Surgery, Chelsea and Westminster Hospital, London, United Kingdom; b Department of Surgery and Cancer, Imperial College, London, United Kingdom; c Department of Colorectal Surgery, Royal Marsden Hospital, London, United Kingdom

**Keywords:** Lower gastrointestinal bleeding, Balloon tamponade, Sengstaken-Blakemore tube, Case report

## Abstract

**Introduction:**

Acute lower gastrointestinal haemorrhage can potentially be life-threatening. We present a case of a massive rectal bleed which was managed successfully with a balloon tamponade device designed for upper gastrointestinal haemorrhage.

**Presentation of case:**

A 75-year-old gentleman, with a history of human immunodeficiency virus and cirrhosis with portal hypertension, presented with bright red rectal bleeding. Investigations showed a low haemoglobin level (74 g/L) and deranged clotting. Oesophago-gastro-duodenoscopy demonstrated no fresh or altered blood. Flexible sigmoidoscopy revealed active bleeding from a varix within the anterior rectal wall 4 cm from the anal verge. Efforts to stop the bleeding, including endoscopic clips, adrenaline injection and rectal packing, were unsuccessful and the patient became haemodynamically unstable. A Sengstaken-Blakemore tube was inserted per rectum and the gastric balloon was inflated to tamponade the lower rectum. The oesophageal balloon was then inflated to hold the gastric balloon firmly in place. A computed tomography angiogram demonstrated no evidence of haemorrhage with balloon tamponade. After 36 h, the balloon was removed with no further episodes of bleeding.

**Discussion:**

The application of a balloon tamponade device should be considered in the management algorithm for acute lower gastrointestinal bleed. Advantages include its rapid insertion, immediate results and ability to measure further bleeding after the catheter has been placed.

**Conclusion:**

Sengstaken-Blakemore tube per rectum may effectively control massive low rectal bleeding when alternative methods have been unsuccessful.

## Highlights

• Acute lower gastrointestinal bleeding can potentially be life-threatening.
• Sengstaken-Blakemore tube per rectum may effectively control rectal bleeding.
• Advantages of balloon tamponade include rapid insertion and immediate results.
• This method could be considered in the management algorithm of rectal bleeding.

## 1 Introduction

Lower gastrointestinal (LGI) haemorrhage is defined as bleeding distal to the ligament of Treitz [1], with approximately 90% bleeding originating from the colon [2,3]. The incidence of LGI haemorrhage is 33-87/100,000 [4] and accounts for 3% of emergency surgical referrals [5]. Without prompt diagnosis and intervention, LGI haemorrhage may be life-threatening. Treatment options include: conservative medical therapy (blood transfusion, antifibrinolytic agents and withholding oral anticoagulants); endoscopy; interventional radiology, and surgery. Endoscopic methods, such as injection therapy, clipping or band ligation, endoloops or thermal coagulation, are usually sufficient in arresting the bleeding [[6], [7], [8]]. Radiological embolisation can also be performed using coils, liquid agents or particles. Emergency laparotomy should be reserved for cases where life-threatening bleeding persists despite other interventions.

The application of balloon tamponade devices is well established in the management of upper gastrointestinal and postpartum haemorrhage [[9], [10], [11], [12]]. Tamponade devices, such as the Minnesota tube or Sengstaken-Blakemore tube, have been specifically designed for the management of upper gastrointestinal bleeding from oesophageal varices often in patients who are haemodynamically unstable providing time for definitive intervention. There are limited reports on the use of balloon tamponade devices for the management of LGI haemorrhage [[13], [14], [15], [16], [17], [18]].

Herein we present an emergency case of a continuous rectal bleed in a patient with multiple co-morbidities who was a poor candidate for surgery. The lower GI bleed did not respond to several medical and endoscopic interventions and a decision was made to insert a Sengstaken-Blakemore tube per rectum. We describe our technique of application and show that the balloon tamponade device, if used correctly, can achieve rapid and effective haemostasis, providing an opportunity to rescue the patient from major haemorrhage. This work has been reported in line with the SCARE criteria [19].

## 2 Case presentation

A 75-year-old gentleman presented with a one-week history of bright red rectal bleeding and fever. His past medical history included: history of human immunodeficiency virus, hepatitis C, decompensated cirrhosis with ascites, portal hypertension, transient ischaemic attack, excess alcohol consumption and smoking. He also had severe aortic stenosis, for which he underwent a transcatheter aortic valve implantation, squamous cell carcinoma of the tongue, basal cell carcinoma and depression. His drug history included anti-retrovirals, aspirin, thiamine, vitamin B, folic acid, omeprazole and fluoxetine. On examination, the patient’s abdomen was distended with minimal tenderness. Digital rectal examination revealed active bleeding with no obvious masses or haemorrhoids. Blood investigations showed a haemoglobin level of 74 g/L, platelet count of 54 × 10^9^/L and deranged clotting and liver function tests. A computed tomography (CT) angiogram demonstrated splenomegaly and ascites but did not identify any specific cause for rectal bleeding. He was optimised medically prior to endoscopy with intravenous antibiotics, tranexamic acid, vitamin K, human albumin solution and a transfusion of one unit of packed red blood cells.

Oesophago-gastro-duodenoscopy demonstrated patchy candidiasis and a single varix in the lower third of the oesophagus. There was mild portal hypertensive gastritis in the cardia and fundus of the stomach and the lower body of the stomach with no gastric varices. No fresh or altered blood was seen within the upper gastrointestinal tract. On flexible sigmoidoscopy, there was a visible point of continuous fresh bleeding on the anterior wall of rectum around 4 cm from the anal verge. With the exception of a possible rectal varix there was no overlying mucosal abnormality at this site. Bleeding persisted despite the application of eight resolution clips (Boston Scientific), adrenaline injections and rectal packing. The patient became haemodynamically unstable with a blood pressure of 70/46 mmHg. A massive haemorrhage protocol was activated with immediate transfusion of packed red blood cells, fresh frozen plasma, platelets and terlipressin.

**Fig. 1:**
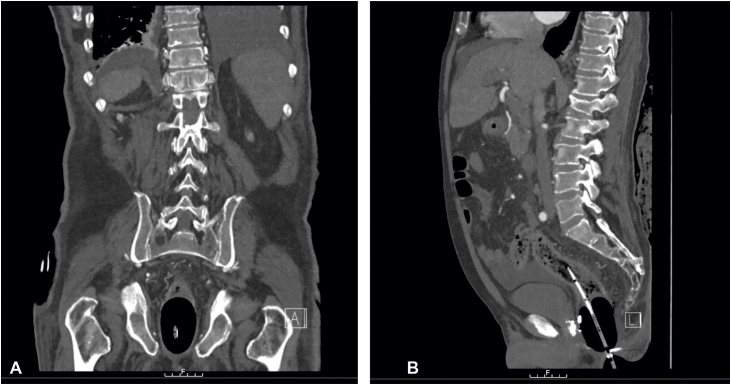
Coronal (A) and sagittal (B) views of computed tomography angiogram (non-enhanced, arterial and portal venous images were obtained) demonstrated no evidence of frank haemorrhage in the gastrointestinal tract. Sengstaken-Blakemore tube placement in the rectum is shown, with inflation of the gastric and oesophageal balloons, which immediately stopped the lower gastrointestinal bleed via balloon tamponade. Endoscopic clips applied during flexible sigmoidoscopy can also be seen.

A decision was made to insert a Sengstaken-Blakemore tube per rectum and to inflate the gastric balloon using 100 mL of air to tamponade the lower rectum. The oesophageal balloon was then inflated (external to the anus) to provide gentle traction to ensure that the balloon was firmly situated in the rectum and exerting optimal pressure onto the bleeding point on the rectal wall. The luminal port was connected to the catheter bag for free drainage and the bleeding stopped immediately. The blood pressure responded and increased to 95/54 mmHg. A subsequent CT angiogram demonstrated no evidence of haemorrhage with the effects of balloon tamponade (Fig. 1). There was no free air noted in the abdomen and no obvious varices in the portal venous phase.

The patient was closely monitored in the intensive care unit following endoscopy where he remained haemodynamically stable. He was further managed with intravenous antibiotics and terlipressin. After 36 h, the haemoglobin level was 92 g/L with a platelet count of 70 × 10^9^/L and the balloon was removed at this point to reduce the risk of pressure necrosis and ulceration of the compressed rectal wall. There were no further bleeding episodes with two months of follow-up.

## 3 Discussion

The use of balloon tamponade devices in the management of LGI haemorrhage is not well established with only a few cases reported in the literature. Table 1 summarises the cases of LGI bleed treated with balloon tamponade present in the literature [[13], [14], [15], [16], [17], [18]]. There are different application techniques, gastric balloon inflation (ranging from 50 mL to 350 mL of air/water) and removal times (ranging from 24 h to 60 h) reported with either interval deflation or complete removal. There were no further episodes of bleeding in all the cases.

**Table 1:** Summary of literature review on the placement of balloon tamponade devices for the management of lower gastrointestinal bleeding. There was successful tamponade in all cases with no further episodes of bleeding reported. **List of abbreviations:** M, male; F, female.

Authors, year	Gender/Age	Presentation of case	Type and inflation extent of balloon	Balloon tamponade time
McGuinness et al. [13], 2004	M/65 years	Bleeding following transanal excision of a large tubovillous adenoma of the rectum	Minnesota tube (50 mL of water in gastric balloon and 100 mL of water in oesophageal balloon)	24 h
Su Min Cho et al. [14], 2006	M/51 years	Bleeding following polypectomy and then rectal varices bleed after laparotomy	Minnesota tube (200 mL of air in gastric balloon)	24 h
Marshall et al. [15], 2007	M/54 years	Bleeding from a stapled ileorectal anastomosis in a patient who previously underwent an emergency total colectomy and defunctioning loop ileostomy	Minnesota tube (200 mL of air in gastric balloon)	60 h
Neeki et al. [16], 2019	M/76 years	Continuous rectal haemorrhage from ulcerated mucosa at the dentate line	Minnesota tube (200 mL of air in gastric balloon and 300 mL of air in oesophageal balloon)	24 h
Roy et al. [17], 1996	F/75 years	Rectal bleeding due to angiodysplasia	Sengstaken-Blakemore tube (350 mL of air in gastric balloon)	48 h
Michopoulou et al. [18], 2013	F/64 years	Massive haemorrhage following rectal biopsies	Sengstaken-Blakemore tube (250 mL of normal saline in gastric balloon)	48 h

Advantages of the Sengstaken-Blakemore or the Minnesota tube are ease of availability, rapid insertion into the rectum and patient acceptability. Benefits over alternative methods, such as packing, include immediate haemostasis and the ability to measure further bleeding after the catheter has been placed. Potential complications of balloon tamponade include ischaemic necrosis, ulceration and perforation of the intestine, particularly in cases of excessive or prolonged inflation [13,20]. To avoid this, close monitoring and gradual deflation in the case of visceral pain is recommended [13]. On the other hand, underfilling of the balloon may lead to migration of the tube proximally [15]. Careful digital palpation of the balloon can confirm that the balloon is optimally sited and pressurised.

## 4 Conclusion

We describe how the application of a Sengstaken-Blakemore tube can control life-threatening bleeding and stabilise the patient during a significant LGI haemorrhage. The technique has proven to be particularly useful when the bleeding point is difficult to locate if the haemorrhage is brisk or there is no raised mucosal lesion found. Thus, the application of a balloon tamponade device should be considered in the management algorithm for acute LGI bleed and can be reserved for when all other alternative methods have failed. It is a simple, safe and effective technique to provide immediate haemostasis especially in patients who are not good candidates for general anaesthesia and surgery.

## Declaration of Competing Interest

All authors declare that there is no financial or personal conflict of interest related to this work.

## Funding

This research did not receive any specific grant from funding agencies in the public, commercial, or not-for-profit sectors.

## Ethical approval

We declare that our institution does not require ethical approval for case reports.

## Consent

Written informed consent was obtained from the patient for publication of this case report and accompanying images. A copy of the written consent is available for review by the Editor-in-Chief of this journal on request.

## Author contribution

MF performed the collection, analysis and interpretation of the data and drafted the manuscript. PB, AH and MI contributed to the analysis of the data and critical revision of the manuscript. PT and CK performed the procedure and approved the final manuscript.

## Registration of research studies

Not applicable.

## Guarantor

Christos Kontovounisios.

## Provenance and peer review

Not commissioned, externally peer-reviewed.
